# Mapping compassion from others: a mixed-methods research protocol on religious and secular compassionate acts and their association with psychological well-being

**DOI:** 10.3389/fpubh.2026.1782870

**Published:** 2026-09-10

**Authors:** Carlo Lazzari, Paul Crawford, Yasuhiro Kotera

**Affiliations:** 1 Institute of Mental Health, University of Nottingham, Nottingham, United Kingdom; 2 Centre for Infectious Diseases Education and Research, The University of Osaka, Osaka, Japan; 3 Department of Social Sciences, Azerbaijan University, Baku, Azerbaijan

**Keywords:** altruism, compassion, compassion from others, prosocial behavior, religiosity, secularism, well-being

## Abstract

**Introduction:**

Compassion is central to moral and relational functioning, yet its determinants and systemic effects remain a subject of debate. The study linked to the current protocol will examine how fear of and openness to compassion from others are associated with well-being, emotional openness, and prosocial behavior. Additionally, it investigates how spirituality, religiosity, and secularism shape interpretations of compassion and altruism among working adults from diverse religious and ethnic backgrounds.

**Methods:**

This research protocol refers to a future study, not started yet, that will employ a concurrent mixed-methods design, combining quantitative and qualitative online survey data with qualitative Microsoft Teams face-to-face interviews to examine how adults interpret compassionate acts through religious, secular, or blended lenses. Biographical and socio-demographic characteristics will be treated as independent variables, psychological outcomes as dependent variables, and received compassion as a moderating variable influencing these associations. Guided by the Social Ecological Model, both strands will be analyzed across individual, interpersonal, organizational, and community levels. Quantitative and qualitative findings will be integrated through joint displays and side-by-side interpretation, enabling statistical patterns to be directly compared with narrative themes and producing a coherent multilevel account of how compassion shapes well-being and relational functioning. The quantitative component will incorporate precise regression-based moderation models, covariate adjustment protocols, and missing data handling, while the qualitative strand will rely strictly on thematic analysis to avoid inappropriate methodological crossover.

**Expected results:**

As this is a research protocol, no assumptions are made regarding the direction, magnitude, or significance of findings. The study to which the protocol refers is designed to examine how individuals interpret acts of compassion received from others through religious, secular, or blended frameworks and whether these interpretations are associated with psychological and relational outcomes. Findings will be interpreted as exploratory and context dependent, with conclusions limited to the study sample and design.

**Discussion:**

Findings of the future study may contribute to future discussions concerning compassion within public health, education, and social care contexts; however, any practical recommendations should be regarded as preliminary and exploratory pending further research using longitudinal and representative designs.

## 1 Introduction

### 1.1 Background

Compassion has emerged as a critical focal point across psychology, organizational studies, and the social sciences, reflecting its established role in fostering emotional resilience, social connection, and personal well-being. Empirical evidence consistently demonstrates that compassion mitigates psychological distress and enhances adaptive coping mechanisms while improving relational functioning across healthcare, education, corporate, and community environments (1–3). Although existing literature predominantly conceptualizes compassion as an outward, prosocial action directed toward others or self, comparatively less empirical attention has been dedicated to understanding how compassion is received, interpreted, accepted, and integrated into personal and social experiences. This trend represents a significant gap in public health literature; individuals exhibit substantial variation in how they evaluate compassionate acts from others, whether they perceive them as trustworthy, and how these experiences subsequently modulate their emotional and relational lives (4).

Interpretations of compassion are also mediated by cultural, spiritual, and secular frameworks. Religious traditions frequently frame compassion as a moral imperative or spiritual duty, whereas secular perspectives tend to emphasize empathy, prosocial behavior, or psychological support (5–7). These interpretive divergences directly affect whether compassion is welcomed, resisted, or misunderstood within communities. For instance, individuals with secular identities may conceptualize compassion primarily as interpersonal solidarity or psychological support, whereas those from religious backgrounds may view it as an expression of spiritual care or divine obligation (8). Elucidating these distinct interpretive frameworks is therefore essential for examining how people interpret acts of compassion from others, how these acts affect their lives, and how the interpersonal dynamics of shared compassion modulate personal, interpersonal, and social well-being (9, 10).

Beyond individual demographics, compassion also operates as a critical systemic variable within organizational life. Empirical research demonstrates that compassionate leadership, supportive workplace cultures, and institutionalized prosocial norms significantly contribute to psychological safety, mitigated burnout, and enhanced team cohesion (11–13). Receiving compassion in professional settings has been shown to fortify trust, elevate morale, and foster healthier interpersonal dynamics (8). However, people’s cultures vary widely in the degree to which compassion is structurally valued or expressed (14). Consequently, people may evaluate compassionate acts differently based on their cultural background, professional role, or religious experiences (12). Unpacking these variations is critical for developing inclusive, effective, and culturally competent public health and organizational practices (15).

Building upon prior research that identified reciprocal empathy and compassion as the foundation of collaborative sustainability, a recent study of 877 US adults positioned the reception of compassion as a personal and interpersonal factor associated with psychological well-being and flourishing (8). The empirical findings demonstrated that receiving compassion from others significantly fosters both personal and social well-being, while enhancing overall psychological resilience (8). This structural integration creates psychologically safe environments and normalizes compassion as a collective force, ultimately driving sustainable, equitable health outcomes across modern, pluralistic societies (8).

### 1.2 Conceptual clarification and construct boundaries

Within the quantitative component, operationalization of received compassion will be aligned with established Compassion Focused Therapy literature, particularly measures assessing fears of compassion from others and openness to receiving compassion. This approach facilitates clearer differentiation between compassion and related constructs such as empathy, kindness, social support, and altruism. To address construct fuzziness and ensure analytical rigor, this study explicitly operationalizes ‘compassion from others’ as a distinct interpersonal process involving an affective awareness of distress coupled with a non-judgmental, warm motivation to alleviate it, distinguishable from generic kindness, empathy, or prosocial duty through its specific focus on threat-regulation and soothing systems (16, 17). Boundaries between related constructs are maintained by measuring specific subscales of fears of and openness to compassion, preventing conceptual slide into general social support (1). Compassion is also conceptualized as an individual’s perception that another person has recognized their suffering, responded with emotional understanding, and demonstrated a genuine intention to alleviate or support them through distress (18). This conceptualization draws on contemporary psychological models that view compassion as a multidimensional process involving sensitivity to suffering, emotional engagement, tolerance of distress, and a commitment to compassionate action (2). Although compassion shares features with several related constructs, it remains conceptually distinct. Empathy involves understanding or sharing another person’s emotional experience but does not necessarily motivate actions to reduce suffering (19, 20). Similarly, kindness and broader prosocial behaviors may involve positive actions toward others without the explicit recognition of suffering that characterizes compassion (21, 22). Social support encompasses emotional, informational, and practical assistance but does not inherently require compassionate awareness or motivation, while altruism refers more broadly to behavior intended to benefit others regardless of whether it arises from compassion or the recognition of distress (23, 24). By focusing specifically on the lived experience of receiving compassion during adversity, the proposed study introduced by the protocol seeks to examine a construct that is theoretically distinct from, although related to, empathy, kindness, social support, altruism, care, and other forms of prosocial behavior (25, 26). This conceptual clarity provides a stronger foundation for the study’s research aims, measurement strategy, and interpretation of findings. Psychological well-being, as reported in the current protocol, refers to an individual’s ability to function positively and effectively in life while experiencing personal growth, meaningful relationships, self-acceptance, autonomy, environmental mastery, and a sense of purpose (27). Rather than focusing solely on happiness or the absence of mental illness, psychological well-being emphasizes the realization of one’s potential and the ability to live a meaningful and fulfilling life (28).

### 1.3 Theoretical framework

Using the Social Ecological Model (SEM) as a conceptual foundation, this study situates compassion within the multiple social contexts in which health and well-being are experienced and understood (29, 30). The SEM is widely used in public mental health to recognize that psychological well-being is associated not only with individual characteristics but also with interpersonal relationships, organizational environments, community contexts, and broader societal conditions (27, 28). Within this framework, experiences of receiving compassion are examined across several ecological levels. At the individual level, compassion may be associated with psychological resilience, emotional regulation, and well-being (31, 32). At the interpersonal level, compassionate interactions among family members, peers, colleagues, and wider social networks are considered in relation to trust, connectedness, and social support (13, 33, 34). Within organizational settings, attention is given to compassionate leadership, workplace culture, psychological safety, and staff well-being (9, 12, 31). At the community level, compassion expressed through civic, cultural, religious, and secular structures is examined in relation to belonging, social cohesion, and collective well-being (31, 35, 36). Finally, at the societal level, broader social norms, public discourse, and cultural understandings of compassion are considered alongside issues of stigma, inclusion, and empathy (37, 38). Consistent with public mental health approaches that emphasize supportive social environments and community resources, the SEM is employed as a heuristic framework for organizing quantitative variables and qualitative themes across ecological levels. It is not used to test causal pathways or cross-level mechanisms, but rather to support a structured interpretation of how compassion and psychological well-being are understood within diverse social contexts.

### 1.4 Knowledge gap

Despite growing academic interest in the construct of compassion, there remains a critical lack of empirical research examining how adults interpret compassion received from others, how these interpretations diverge across religious and secular frameworks, and how the reception of compassion associates with personal and social well-being, social interactions within organizational, multicultural, and multifaith contexts. Existing literature has predominantly focused on compassion training, self-compassion, compassion satisfaction, compassion fatigue, or clinical interventions, leaving a distinct gap in the understanding of compassion from others and its association with psychological well-being (39–41). Furthermore, limited empirical research explores how compassion is understood across multiple ecological layers, and how interpretations at one micro (personal) or macro (interpersonal) systemic level may be associated with experiences at another. While recent studies highlight the importance of compassion within educational and workplace settings, specifically its associations with mental health, motivation, and relational functioning, the precise mechanics underlying the reception of compassion remain significantly underexplored (42, 43). To eliminate conceptual extension, this study narrows its operational scope strictly to the psychological and interpretive correlates of received compassion among working adults, excluding peripheral explorations of macro-level public health policy design, educational curriculum overhauls, and institutional governance structures.

### 1.5 Aims

The overarching aim of the study proposed by the current protocol is to explore how adults of both genders aged 18 years and above interpret acts of compassion from others, with particular attention to whether they draw on religious, secular, or blended frameworks of meaning. The project further seeks to examine how the reception of compassion is associated with personal and interpersonal well-being, emotional functioning and social interactions within organizational and community settings. To achieve this, the proposed study will conceptualize biographical and socio-demographic characteristics, including age, gender, ethnicity, religious affiliation, occupational role and community context, as independent variables that may shape how compassion is understood and interpreted. Psychological outcomes such as general and psychological well-being, flourishing, emotional functioning, relational quality and broader indicators of welfare will be treated as dependent variables. Received compassion will be positioned as a moderating variable that will relate with the strength and direction of associations between these independent and dependent variables, reflecting its potential to buffer or amplify psychological and relational outcomes.

These aims are addressed through a concurrent mixed-methods design that will integrate quantitative and qualitative data to capture both measurable patterns and rich interpretive detail. Quantitative analyses will examine the associations between independent variables, the moderating association of received compassion and the resulting psychological outcomes, while qualitative data will provide contextual depth by illuminating the interpretive frameworks through which participants understand compassionate acts from others. The integration of both strands during interpretation will ensure that the analytical strategy will remain fully aligned with the study aims and variable structure, consistent with established mixed-methods guidance (44, 45).

## 2 Methods

### 2.1 Study design and scope

The proposed research project will adopt a concurrent mixed-methods design in which quantitative and qualitative data will be collected simultaneously and integrated during analysis to generate a comprehensive understanding of how adults interpret compassion from others. The proposed protocol also aligns with STROBE (Strengthening of Reporting of Observational Studies in Epidemiology) statement (Table 1) (46).

**Table 1:** STROBE checklist for the current research protocol.

STROBE item	Description	Reported?	Location in protocol
Title_and_abstract	Indicate study design in title/abstract	☑	Title states *“Mixed-Methods Research Protocol”*; abstract describes design.
Background_rationale	Scientific background and rationale	☑	Background section: *“Compassion has emerged as a critical focal point…”*
Objectives	State specific objectives	☑	Aims, Research Questions, Hypotheses.
Setting	Describe setting, locations, dates	☑	Online survey + Microsoft Teams interviews; organisational, religious, secular contexts.
Participants	Eligibility criteria, selection methods	☑	Adults ≥18; must have experienced compassion; opportunistic + snowball sampling.
Variables	Define all variables	☑	Independent, dependent, moderating variables clearly defined.
Data_sources_measurement	Sources and methods of measurement	☑	Survey (quantitative + qualitative), interviews, validated compassion scales.
Bias	Address potential sources of bias	☑	Self-selection bias acknowledged; non-probability sampling limitations.
Study_size	Explain study size	☑	Non-probability sampling; no power calculation.
Quantitative_variables	Handling of quantitative variables	☑	Moderation models, covariates, reliability testing.
Statistical_methods	Statistical methods	☑	PROCESS moderation, descriptive statistics, missing data handling.
Participants_numbers	Numbers at each stage	□ (Future study)	Will be reported after data collection.
Descriptive_data	Characteristics of participants	□ (Future study)	To be reported post-study.
Outcome_data	Outcome data	□ (Future study)	No results yet.
Main_results	Main results	□ (Future study)	Protocol states: *“no assumptions are made….”*
Other_analyses	Subgroup/sensitivity analyses	☑	Secondary exploratory analyses described.
Key_results	Key results summary	☑ (Protocol-level)	Expected Results section: exploratory, non-directional.
Limitations	Study limitations	☑	Non-probability sampling, cross-sectional design, no causal inference.
Interpretation	Interpretation	☑	*“Exploratory and context dependent….”*
Generalisability	External validity	☑	Transferability limited to similar contexts.

The proposed research will be an observational cross-sectional study with assessment of the population at one point in time. This approach will enable the future study to examine how religious, secular, and blended ethical frameworks shape perceptions of compassion and how these interpretations relate to intrapersonal and interpersonal well-being. Biographical and socio-demographic characteristics, including age, gender, ethnicity, religious affiliation, occupational role, and community context, will be treated as independent variables, while psychological outcomes, emotional functioning, relational quality, and broader indicators of well-being serve as dependent variables. Received compassion will be conceptualized as a moderating variable that shapes the strength and direction of associations between sociodemographic variables and psychological outcomes, and this moderating role will be explored through both quantitative and qualitative analysis.

Quantitative data collection will be structured around a mixed-methods survey instrument that combines multiple-choice quantitative items with open-ended qualitative questions, enabling the capture of both measurable patterns and narrative descriptions of compassionate experiences. To deepen contextual understanding, qualitative semi-structured and face-to-face interviews will be conducted and audio-video-recorded via Microsoft Teams. These interviews will allow participants to elaborate on the meanings, contexts and interpretive frameworks through which they understand compassion from others, and to describe how receiving and accepting compassion affects their emotional and relational functioning. Inclusion criteria will specify adults aged 18 years and above from any cultural, religious or social background, including those affiliated with religious or secular organizations. Participants must have experienced compassion from others in their daily lives and be able to reflect on compassionate responses received during periods of illness, personal tragedy, adversity or significant life challenges. This will ensure that the proposed study will capture a broad spectrum of compassionate encounters across both daily and high-stress contexts.

Integration of quantitative and qualitative data will occur throughout an interpretive process. Joint displays will be used to align statistical patterns, such as correlations, cross-correlations, and group differences, with qualitative themes derived from interview narratives, enabling direct comparison between numerical trends and lived experiences. Side-by-side interpretation will further support integration by using quantitative findings to identify areas requiring deeper qualitative explanation, while qualitative insights contextualize and refine the interpretation of statistical associations. The Social Ecological Model will provide an overarching framework for this integration, allowing both strands to be interpreted across individual, interpersonal, organizational, and community levels. This multilevel structure will support a coherent synthesis of how compassionate acts are understood and how they are associated with well-being and relational functioning. By allowing both strands to contribute equally to the final interpretation, the proposed study aligns with established methodological guidance in mixed-methods research, thereby strengthening the explanatory power and conceptual clarity of the findings (44, 45). Recognizing the design limitations of non-probability, cross-sectional research, causal inferences are strictly avoided; the methodology is explicitly tailored to explore associations, moderation effects, and subjective interpretations rather than establish broad explanatory mechanisms across ecological levels.

### 2.2 Research questions (Q) and hypotheses (H)

#### 2.2.1 Research questions (Q)

Research questions using the *PI/ECOS* (*P*opulation, *I*ntervention/*E*xposure, *C*omparison, *O*utcomes, *S*ettings) framework, tailored for the present research protocol, are as follows (47):

*Q1:* Among adults aged 18 years and above, what are the associations between sociodemographic and biographical characteristics, including age, gender, ethnicity, religious affiliation, occupational role, and community context (independent variables), and psychological well-being (primary outcome), and to what extent are these associations moderated by the degree to which individuals receive and accept compassion from others (moderating variable) across secular, religious, and organizational settings?

*Q2:* Among adults aged 18 years and above, how do individuals interpret and make sense of compassionate acts received from others, and to what extent are these interpretations understood through religious, secular, or blended frameworks across different social and organizational contexts?

#### 2.2.2 Research hypotheses (H)

The quantitative hypothesis aligns with Q1:

*H₁:* Among adults aged 18 years and above, the association between sociodemographic and biographical characteristics and psychological well-being is significantly moderated by the degree to which individuals receive and accept compassion from others across secular, religious, and organizational settings. For analytical clarity, psychological well-being represents the primary quantitative outcome variable and received compassion represents the primary moderating construct examined within regression-based analyses. Additional analyses involving other psychological and demographic variables will be considered secondary and exploratory.

*Null Hypothesis (H₀₁):* The degree to which individuals receive and accept compassion from others does not significantly moderate the association between sociodemographic and biographical characteristics and psychological well-being among adults across secular, religious, and organizational settings.

For the qualitative component associated with Q2, no formal hypothesis is proposed, consistent with the exploratory nature of qualitative inquiry. Instead, qualitative analysis seeks to explore participants’ interpretations and experiences of receiving compassion and the meanings attributed to these experiences within religious, secular, and blended frameworks.

### 2.3 Sampling

An opportunistic sampling approach will be used to identify participants who are readily accessible and willing to take part, drawing on individuals across a range of settings including employees within organizations, online survey respondents, clients or service users, members of religious communities and attendees of secular organizations. To extend recruitment beyond these immediately reachable groups, the future study introduced by the current protocol will incorporate snowball sampling, whereby initial participants will be invited to share study information within their personal or professional networks. This participant-led referral process will enable the identification of additional eligible respondents who may not be directly accessible through organizational or community channels. The combined use of opportunistic and snowball sampling reflects the exploratory aims of the proposed study and the need to access diverse experiences of compassion across multiple social contexts. As a non-probability strategy, this approach does not support statistical generalizability; however, it is well suited to capturing variation in lived experience and generating rich qualitative and quantitative insights into how compassion is interpreted and received (48). Acknowledging that this non-probability sampling strategy will introduce self-selection bias, favoring individuals already interested in spirituality, mental health, or compassion, the outcome of the proposed study will make no claims regarding statistical generalizability or population-level prevalence. Findings will be interpreted strictly as reflections of the participating convenience cohort, with transferability limited to analytically comparable demographic contexts.

### 2.4 Type of data collected

The proposed study will employ a mixed-methods design that will integrate quantitative online survey data with qualitative interview data, as well as qualitative responses to the open-ended items within the survey.

#### 2.4.1 Mixed method integration

A convergent mixed-methods design will be adopted, whereby quantitative and qualitative data will be collected during a similar timeframe, analyzed independently, and subsequently integrated to generate inferences that draw upon both datasets (49). This design is particularly appropriate where a phenomenon is best understood through the combination of numerical trends and participants’ subjective experiences, allowing researchers to obtain a more comprehensive understanding than could be achieved through a single methodological approach alone (40, 50). In the proposed study, quantitative data will identify patterns, associations, and potential moderation effects between key variables, whereas qualitative data will provide explanatory depth by exploring how participants interpret and make meaning of experiences of receiving compassion from others.

Consistent with recommendations for convergent designs, quantitative and qualitative datasets will initially be analyzed separately using procedures appropriate to each methodological tradition to preserve the integrity of each strand prior to integration (40). Following separate analyses, integration will occur using joint displays, which provide a structured mechanism for visually and conceptually comparing findings across datasets and facilitating the development of meta-inferences (41). Joint displays will align statistical findings, thematic patterns, and representative participant accounts to enable systematic examination of points of agreement and disagreement.

Integration will focus on the assessment of convergent, complementary, and divergent findings. Convergent findings, where quantitative and qualitative results support similar conclusions, will enhance the credibility and robustness of interpretations through triangulation. Complementary findings, where one dataset expands, enriches, or contextualizes the other, will be used to provide a fuller understanding of the phenomenon under investigation. Divergent findings, where discrepancies emerge between datasets, will not be regarded as methodological weaknesses but as opportunities to explore contextual influences, subgroup differences, alternative explanations, or dimensions of experience not captured by a single method alone (40, 41).

Equal priority will be afforded to both the quantitative and qualitative strands, with each contributing distinct yet complementary forms of evidence toward addressing the research aims. The final stage of analysis of the proposed research will involve the interpretation of integrated findings and the generation of overarching inferences that synthesize insights from both strands and provide a richer, more nuanced understanding of the experience and impact of receiving compassion from others (40, 41, 46).

#### 2.4.2 Quantitative analysis plan and moderation strategy

Quantitative data will be collected remotely through secure, University-approved online survey platforms, for example SurveyMonkey, using closed-ended items to generate measurable indicators of compassion from others and open-ended questions to capture participants’ personal accounts in their own words. To rectify previous ambiguities in statistical execution, quantitative data will be analyzed using a structured hierarchical framework:

1 Preliminary Testing: Data screening will assess normality, linearity, and multicollinearity. Scale reliability will be assessed using Cronbach’s alpha coefficients for all multi-item measures. Values of 0.70 or higher will generally be considered acceptable indicators of internal consistency. Measures demonstrating inadequate reliability will be examined and reported transparently before proceeding with regression modeling. Missing data will be handled via listwise or pairwise deletion depending on missingness patterns, with Little’s MCAR test applied where appropriate (51).
2 Primary Analyses: Descriptive statistics, correlation coefficients, and chi-square tests of independence will map baseline associations.
3 Moderation Analysis: To test the core moderation hypothesis (H₁), regression-based moderation models (utilizing PROCESS macro or equivalent structural equation modeling frameworks) will be executed, incorporating mean-centering for continuous predictors and controlling for relevant sociodemographic covariates (e.g., age, gender, baseline stress) (52). Corrections for multiple comparisons (e.g., Bonferroni adjustments) will be applied to control Type I error rates across omnibus tests.

#### 2.4.3 Qualitative methodology and thematic rigor plan

The qualitative component will also comprise synchronous, semi-structured audio-video recorded interviews conducted remotely via MS Teams or other University of Nottingham-approved video-conferencing software. These interviews will be audio or video recorded according to participants’ preference and will follow a flexible interview schedule designed to explore meanings, interpretations, and contextual associations about experiences of receiving compassion from others. Where appropriate and feasible, optional face-to-face interviews may also be undertaken to support deeper exploration of complex or sensitive themes. All data will be stored using encrypted, University-approved electronic systems with access restricted to the principal investigator and supervisory team. Primary interview transcripts will be analyzed exclusively using established inductive thematic analysis (Braun and Clarke), focusing on semantic and latent codes regarding participants’ interpretive frameworks (53).

### 2.5 Sample size calculation and statistical power plan

The sample size calculation will be designed to ensure adequate statistical power for the primary quantitative analyses, particularly the proposed regression-based moderation models. Statistical power analyses will be conducted using G*Power, adopting the conventional criteria of 80% statistical power (1-β = 0.80) and a significance threshold of α = 0.05 (54, 55). Given that the principal hypothesis concerns moderation effects, sample size estimation will be based on multiple linear regression models incorporating the main predictor, moderator variable, interaction term, and relevant sociodemographic covariates.

Consistent with recommendations for behavioral and social science research, calculations will assume the detection of small-to-medium effect sizes, recognizing that interaction effects in moderation analyses are often smaller and more difficult to detect than main effects (56, 57). Preliminary power analyses indicate that a sample of at least 500 participants will provide adequate power to detect small-to-medium effects while accommodating the anticipated complexity of the regression models and potential participant attrition or incomplete responses. The proposed sample size also exceeds minimum recommendations for stable estimation of regression coefficients in multivariable analyses. Additional quantitative analyses, including descriptive statistics, correlations, chi-square tests, and group comparisons, will be conducted to explore relationships among variables and characterize the sample. Effect sizes will be reported alongside significance tests to facilitate interpretation of practical significance. Where appropriate, chi-square effects will be expressed using Cohen’s *w* and group differences using standardized effect size indices (58, 59).

For the qualitative component, sample adequacy will be determined according to the principle of information power and thematic saturation. Previous methodological research suggests that saturation is frequently achieved within approximately 12 to 20 interviews, although the precise number depends upon the scope of inquiry, participant heterogeneity, and complexity of the phenomenon under investigation (60, 61). As the proposed research will adopt a mixed-methods design, quantitative and qualitative sample size decisions will be guided by different methodological principles. Quantitative sampling will prioritize statistical precision and power, whereas qualitative sampling will prioritize depth, richness, and conceptual completeness (41). Importantly, statistical power calculations will not overcome the inferential limitations associated with non-probability sampling. Although power analysis will enhance the likelihood of detecting effects within the recruited sample, findings will not be interpreted as fully representative of the wider population without consideration of potential sampling bias.

### 2.6 Participants recruitment

Potential participants will be recruited through a combination of opportunistic and snowball sampling to ensure broad inclusion across organizational, community, religious, and secular contexts. Individuals aged eighteen years and above who have experienced compassion from others will be identified through online networks, professional forums, community groups, and social media, as well as through organizational and community gatekeepers such as team leaders, administrators, and religious or secular community representatives, who will distribute the study information without knowing who subsequently chooses to participate. Interested individuals will self-select into the study by accessing the online survey or contacting the research team directly, and initial participants will be invited to share the study information within their personal and professional networks to extend recruitment to otherwise inaccessible groups. Eligibility screening will occur at the point of contact, after which participants will receive a participant information sheet and provide informed consent before completing the mixed-methods online survey and, where applicable, a Microsoft Teams interview. Recruitment will continue until sufficient variation in demographic and experiential characteristics is achieved, with all participation voluntary and withdrawal permitted at any point prior to data analysis.

### 2.7 Sampling limitations and generalizability plan

The proposed research will employ convenience and snowball sampling methods, which may limit the representativeness of the sample and introduce self-selection bias. Individuals who choose to participate will probably differ systematically from those who do not, potentially influencing the observed patterns of responses. Consequently, although the proposed sample size is intended to ensure adequate statistical power for detecting hypothesized relationships within the study sample, the findings will not be interpreted as statistically representative of the wider population. Rather, the results will be considered in relation to their transferability to similar contexts and populations, with appropriate caution regarding broader population-level inferences.

### 2.8 Statistical software and analysis plan

Statistical analyses will be conducted using MedCalc software for both descriptive and inferential procedures, while sample size estimation and statistical power will be calculated using G*Power, and effect sizes computed through Psychometrica (49, 54, 62). Model fit will be evaluated using the Chi-square Goodness of Fit Index (GFI) following Statology guidance, and the power of chi-square tests will be estimated using the Statistic Kingdom calculator (63, 64). Quantitative analyses will include examining closed ended responses through percentages, correlation coefficients, chi-square tests, to determine statistical significance. Qualitative analysis will be undertaken using QDA Miner and QDA Simstat, applying reflexive thematic analysis to identify recurring meanings and patterns within participants’ accounts (53, 65). The qualitative component involves the analysis of primary interview data using thematic analysis. Thematic development will focus on participants’ lived experiences and interpretive frameworks regarding compassion from others.

### 2.9 Measurement instrument

#### 2.9.1 Qualitative survey instrument plan

Qualitative data will be collected through face-to-face, semi-structured, video-recorded interviews designed to explore participants’ experiences and interpretations of receiving compassion from others. Interviews will be conducted via TEAMS. The interview schedule comprises 22 open-ended questions organized into nine domains: demographic context, experiences of receiving compassion, interpretations of compassion, attitudes toward receiving compassion, psychological well-being and resilience, settings in which compassion is experienced, organizational and social dimensions of compassion, the relationship between secular and religious interpretations of compassion, and closing reflections. The semi-structured format will provide a consistent framework across interviews while allowing participants the flexibility to elaborate on personally meaningful experiences and perspectives. Attention will be given to understanding how individuals interpret compassionate acts within religious, secular, or blended frameworks and how these interpretations relate to well-being, resilience, social connectedness, and organizational life. Follow-up prompts will be used to encourage detailed reflection, clarify meanings, and explore contextual factors shaping participants’ experiences. The interview guide was developed to align directly with the study’s research questions and conceptual framework, facilitating an in-depth exploration of the subjective meanings, contextual influences, and relational dimensions associated with receiving compassion from others. Qualitative findings will complement the survey data by providing rich narrative accounts that help contextualize quantitative patterns and identify themes that may not be adequately captured through structured questionnaire items alone (Supplementary Appendix). The qualitative interview schedule was developed through an iterative process informed by existing compassion theory, validated compassion measurement frameworks, and findings from prior empirical and evidence-synthesis research conducted by the research team. Conceptually, the interview guide was informed by Compassion Focused Therapy literature and contemporary multidimensional models of compassion that emphasize sensitivity to suffering, emotional engagement, and action-oriented responses to distress, and our former studies on the topic (66–69). Several domains will be explored within the interview schedule, particularly participants’ openness to receiving compassion, interpretations of compassionate acts, emotional responses to receiving compassion, and perceived effects on well-being, as informed by constructs examined in the Fears of Compassion Scales and the Compassionate Engagement and Action Scales (70, 71).

Development of the interview questions was also informed by the authors’ previous empirical investigation of compassion in the general adult population, which examined religious and secular interpretations of compassionate acts within social relationships and organizational settings (8). Findings from that study highlighted the importance of exploring how individuals make sense of compassionate experiences through diverse religious, spiritual, secular, and blended frameworks and how these interpretations relate to perceptions of well-being, belonging, interpersonal trust, and social connectedness. Consequently, several interview domains were specifically designed to explore these interpretive processes in greater depth.

The interview schedule will be further informed by a recently published systematic review protocol examining the psychosocial effects of receiving compassion from others and its interpretation through religious and secular frameworks (7). This body of work identified recurring themes across the literature concerning the reception of compassion, perceived barriers to accepting compassion, social and cultural influences on compassionate experiences, and associations between received compassion and psychological well-being. These findings will contribute to the selection of interview topics relating to resilience, emotional functioning, organizational experiences, and meaning-making processes.

Accordingly, the interview guide is best understood as a theoretically informed, study-specific qualitative instrument developed from established compassion research, emerging evidence concerning compassion from others, and previous work undertaken by our research team. The purpose of the schedule is not to test predetermined hypotheses but to facilitate an in-depth exploration of how adults understand, experience, interpret, and integrate compassionate acts received from others across diverse religious, secular, organizational, and community contexts.

### 2.10 Quantitative survey instrument plan

Psychological well-being serves as the primary quantitative outcome variable in all moderation analyses. Quantitative data will be collected through an anonymous online questionnaire administered via SurveyMonkey. The survey was specifically developed for the study linked to the protocol to investigate experiences of receiving compassion from others, the meanings attributed to such experiences, and their perceived relationships with psychological well-being, resilience, interpersonal functioning, and organizational life. The instrument comprises a combination of demographic questions, multiple-choice items, Likert-type rating scales, and open-ended questions, allowing both descriptive and exploratory analysis. The questionnaire is organized into several domains, including demographic characteristics, frequency of receiving compassion, sources of understanding compassion, attitudes toward receiving compassion, perceived contributions of compassion to quality of life and well-being, settings in which compassion is experienced, organizational implications of compassion, and the interpretation of compassion through religious, secular, or blended frameworks. Response formats include categorical options, five-point frequency and agreement scales, and free-text narrative responses. This mixed-format design will enable the collection of both quantitative indicators and contextual qualitative data, facilitating a comprehensive examination of how compassion from others is experienced and understood across diverse populations and settings. The survey content was developed in direct alignment with the study’s research questions and conceptual framework and is intended to identify patterns of association between participant characteristics, experiences of receiving compassion, and self-reported indicators of psychological and relational well-being. Open-ended responses will also provide contextual information to support the interpretation of quantitative findings and inform subsequent qualitative analyses. Where appropriate, validated psychometric measures will be incorporated to assess openness to compassion, fears of compassion, and psychological well-being. Internal consistency reliability will be examined for all multi-item measures prior to inferential analysis using Cronbach’s alpha coefficients. Reliability estimates will be reported alongside descriptive statistics to support the interpretability of study findings (Supplementary Appendix).

The quantitative survey was developed using a combination of established compassion and quality-of-life frameworks alongside study-specific items designed to address the research objectives. Items relating to the experience of receiving compassion from others were informed by the Compassionate Engagement and Action Scales (CEAS) and the Fears of Compassion Scales developed within Compassion Focused Therapy research (72, 73). In particular, the survey design drew upon constructs relating to compassion from others, openness to receiving compassion, and potential resistance to compassionate engagement, as operationalized in the work of Asano et al. (70, 72) These studies demonstrated acceptable internal consistency, construct validity, and test–retest reliability for measures assessing compassion from others and fears of receiving compassion. Rather than reproducing these psychometric scales in their entirety, selected items and underlying constructs will be adapted to explore participants’ subjective experiences and interpretations of receiving compassion within religious, secular, and blended frameworks. This approach is considered appropriate given the exploratory mixed-methods nature of the study and the specific emphasis on meaning-making and interpretation. Items assessing psychological well-being and quality of life were informed by the conceptual framework underpinning the World Health Organization Quality of Life instruments (74, 75). The WHOQOL instruments conceptualize quality of life as an individual’s perception of their position in life within their cultural and value context and assess domains including psychological well-being, social relationships, environmental factors, and overall life satisfaction. Survey questions relating to emotional well-being, coping, social functioning, and perceived quality of life will therefore be adapted from these domains to provide indicators of subjective well-being relevant to the study aims. The resulting questionnaire should therefore be regarded as a study-specific instrument informed by validated theoretical and psychometric foundations rather than as a direct replication of any single existing scale. Internal consistency reliability will be evaluated for all multi-item measures using Cronbach’s alpha coefficients prior to inferential analyses, and reliability statistics will be reported alongside descriptive findings.

### 2.11 Ethical considerations

The proposed research will adhere to the University of Nottingham Code of Research Conduct and Research Ethics, the UK Policy Framework for Health and Social Care Research, the principles of the Declaration of Helsinki, and the Belmont Report (76–79). Ethical safeguards will ensure autonomy, confidentiality, inclusivity, and minimal risk. Data will be collected through an anonymous SurveyMonkey questionnaire that does not record names, email addresses, or IP addresses, and uses encryption and secure servers to protect both quantitative and qualitative responses (80). Participants will receive a digital Participant Information Sheet and provide electronic opt-in consent, confirming voluntary participation and the right to withdraw prior to submission. All data will be stored on encrypted University servers in accordance with UK GDPR (General Data Protection regulation), with only aggregated findings reported (81). Risks are minimal, and optional signposting to mental health resources will be provided. Recruitment materials will be culturally and linguistically inclusive.

## 3 Expected results

The future study introduced by this protocol is not yet underway; therefore, no assumptions are made regarding the direction, magnitude, or statistical significance of any quantitative or qualitative findings. Consistent with the protocol, results will be interpreted as exploratory, descriptive, and context-dependent rather than predictive or generalizable. As stated in the manuscript, “As this is a research protocol, no assumptions are made regarding the direction, magnitude, or significance of findings”. Quantitatively, it is expected that the dataset will reveal measurable associations between sociodemographic variables and psychological well-being, with received compassion functioning as a potential moderating variable within regression-based models. These analyses may identify patterns of association, subgroup differences, or moderation effects; however, any observed relationships will be interpreted strictly as correlational. The protocol emphasizes that the study aims to “examine how individuals interpret acts of compassion… and whether these interpretations are associated with psychological and relational outcomes”, without implying causal pathways.

Qualitatively, it is anticipated that participants will describe diverse interpretations of compassion from others, shaped by religious, secular, or blended frameworks. Interview narratives may illuminate how compassionate acts are understood within personal, interpersonal, organizational, and community contexts. These themes will provide contextual depth to quantitative patterns, enabling a multilevel ecological interpretation aligned with the Social Ecological Model. Integrated mixed-methods results are expected to produce convergent, complementary, or divergent findings. Convergent findings may show alignment between statistical associations and narrative accounts; complementary findings may expand or contextualize quantitative trends; and divergent findings may highlight interpretive complexity or subgroup variation. As noted in the protocol, findings will be “interpreted as exploratory and context dependent, with conclusions limited to the study sample and design”.

## 4 Discussion

The expected outcomes of the study are framed as tentative, hypothesis-driven insights that reflect the exploratory nature of the design. Rather than anticipating broad systemic or policy-level effects, the project aims to generate early indications of how adults interpret compassion from others through religious, secular or blended frameworks, and how these interpretations may relate to emotional and relational functioning across different life contexts. Within clinical practice, the findings may offer preliminary insights relevant to therapeutic formulations, particularly in situations shaped by trauma, shame or relational fear. Existing literature suggests that compassion from others is associated with affect regulation and reduced threat-based responding (82, 83). The study proposed by the current protocol seeks to explore how participants understand and experience receiving compassion, and whether these experiences are associated with perceptions of personal well-being, emotional support, safety, and interpersonal connection once compassion is accepted. Such findings may contribute to a broader understanding of received compassion within therapeutic and social relationships, consistent with previous research examining associations between compassion from others, psychological resilience, and social safeness (84–86). Furthermore, by examining these experiences across religious, secular, and blended interpretive frameworks, the study may provide contextually grounded insights relevant to public mental health, well-being, and social connectedness in diverse populations (2, 87). Any practical implications emerging from the study should be considered preliminary and hypothesis-generating. Further longitudinal, experimental, and population-representative research would be required before drawing conclusions regarding causality, effectiveness, policy relevance, or large-scale implementation within healthcare, organizational, educational, or community settings (69, 88).

### 4.1 Strengths and limitations

The proposed study presents several strengths and limitations that reflect the methodological choices underpinning its mixed-methods design. A key strength lies in the combination of a cross-sectional correlational survey with qualitative interviews, enabling both broad empirical mapping and in-depth interpretive exploration of how compassion is received across secular and religious contexts. The qualitative component will strengthen the study by providing rich, contextualized accounts that illuminate the interpretive frameworks through which compassion is understood. Although qualitative findings are not statistically generalizable, they will enhance understanding of processes and experiences across similar contexts. The integration of quantitative and qualitative findings supports a more comprehensive interpretation of the phenomenon under investigation by combining numerical patterns with experiential accounts. The proposed mixed-methods approach aims to strengthen the credibility and contextual richness of the findings while acknowledging the inherent limitations of non-probability sampling and cross-sectional research. An additional limitation arises from the eligibility criterion requiring participants to report previous experiences of receiving compassion. This may preferentially recruit individuals who are more aware of, receptive to, or interested in compassionate experiences, potentially limiting the range of perspectives captured.

However, several other critical limitations must be transparently acknowledged:

- Causality and Temporal Ambiguity: The cross-sectional, single-timepoint design precludes causal inferences; observed associations between received compassion and well-being could operate bidirectionally or be driven by unmeasured confounding variables.
- Sampling Bias: Non-probability convenience and snowball sampling restrict the sample to self-selecting, motivated participants, limiting external validity and preventing population-level generalization.
- Methodological Constraints: Reliance on self-report measures introduces potential recall and social desirability biases, which will be discussed transparently in future reporting.

An additional limitation concerns the cultural and contextual variability inherent in concepts such as religion, spirituality, and secular identity (89). These constructs are complex, multidimensional, and interpreted differently across cultural, social, and personal contexts (90). For some individuals, spirituality may be closely linked to organized religious traditions, whereas for others it may reflect broader sources of meaning, connection, or personal values independent of religious affiliation. Similarly, secular identities are heterogeneous and may encompass diverse philosophical, ethical, and worldview perspectives (91). Furthermore, religious, spiritual, and secular identities are not always discrete or mutually exclusive categories; many individuals hold blended, evolving, or context-dependent identities that draw upon elements of multiple traditions, beliefs, or value systems (34, 92). Consequently, participants may differ in how they understand and self-identify these categories, potentially influencing both their experiences of compassion and their responses to study measures (93, 94). While the mixed-methods design will help capture some of this complexity through qualitative exploration, findings should be interpreted with recognition that meanings associated with religion, spirituality, and secularity may vary across cultural and contextual settings.
