# Supplementary material for: Mapping compassion from others: a mixed-methods research protocol on religious and secular compassionate acts and their association with psychological well-being
Source: Front Public Health. 2026 Sep 10;14:1782870. doi: 10.3389/fpubh.2026.1782870 (PMC13604936; doi:10.3389/fpubh.2026.1782870)
Supplement: Supplementary file 1 [file Supplementary_file_1.docx]

Appendix

# 1 Part A. Qualitative Interview Schedule

1. Opening and Demographic Context (Survey Section A)

These questions are brief and conversational, used solely to contextualize the participant’s narrative.

1. To begin, could you tell me a little about yourself: your age group, gender identity, and educational background?
2. Can you describe your current setting of practice?
   - (Secular, religious, organizational, mixed, or other)
3. Do you identify with any religious or spiritual tradition, or none?
4. What sector do you currently work in, and how long have you been in that environment?

2. Experiences of Receiving Compassion (Survey Section B)

5. In your everyday life, do you feel you receive compassion from others?
   - Can you share a recent example that stands out to you?
6. How often would you say you receive compassion from others?
   - What factors seem to influence how often you experience it?
   - Are there situations where compassion feels more or less likely to occur?

3. Interpretation of Compassion (Survey Section C)

7. When you think about compassion from others, where do your ideas or expectations come from?
   - For example: religious teachings, secular ethics, family upbringing, cultural norms, workplace values, or a mix.
   - Could you share a personal example that illustrates this?
8. In your experience, who decides whether an action counts as compassion?
   - Is it mainly you as the receiver, the person offering the act, both, or sometimes even a bystander?
   - Can you describe a situation where people interpreted an act differently? How was that resolved?

4. Attitudes Toward Receiving Compassion (Survey Section D)

9. How comfortable do you feel when others show you compassion?
   - What makes it easy or difficult to accept?
10. Have you ever felt uneasy, fearful, or resistant when someone tried to show you compassion?
    - What do you think lies behind that feeling?
11. When someone shows you compassion, what emotions tend to arise for you?
    - Gratitude, empowerment, discomfort, undeservingness, or something else?
    - Can you recall a moment that captures this?

5. Impact on Well‑Being and Resilience (Survey Section E)

12. How does receiving compassion from others affect your psychological or emotional well‑being?
    - In what ways does it help, or not help, you?
13. Can you describe how compassion from others influences your resilience when facing challenges?
    - Is there a moment when compassion made a difference in how you coped?
14. Thinking more broadly, how much does compassion from others contribute to your overall quality of life?
    - What aspects of life does it influence most?

6. Settings of Compassion (Survey Section F)

15. In which areas of your life do you most often experience compassion from others?
    - Family, friends, workplace, religious community, society, online, or elsewhere?
    - Why do you think this setting matters most for you?

7. Organizational and Social Impact (Survey Section G)

16. From your perspective, how does receiving compassion from others influence your workplace or organizational environment?
    - For example: teamwork, morale, trust, productivity, or conflict resolution.
    - Can you share an example where compassion had a noticeable organizational effect?

8. Integrative Questions (Linking Secular–Religious Frameworks to Interpretation)

These are added to strengthen your core research aim.

17. When you receive compassion, do you interpret it through a secular lens, a religious/spiritual lens, both, or does it depend on the situation?
    - What shapes that interpretation?
18. Do you feel that secular and religious frameworks of compassion overlap, conflict, or complement each other in your experience?
    - Could you describe a moment that illustrates this?
19. How do your beliefs—whether secular, religious, spiritual, or mixed—shape the meaning you attach to compassion from others?
20. If you think of compassion as a relational experience, what makes it feel authentic, trustworthy, or meaningful to you?

9. Closing Reflection

21. Is there anything else about receiving compassion from others that you feel is important to share?
22. What do you think researchers, organizations, or society often misunderstand about compassion?

## 2 Part B: Online Survey on Receiving Compassion from Others and Its Relationship with Well-Being

The items presented below represent the study-developed component of the questionnaire. Additional validated psychometric measures assessing fears of compassion, openness to compassion, and psychological well-being will be administered alongside these items and analysed according to published scoring procedures.

# 3 Section A. Demographic Information

*Age*

☐ 18–24

☐ 25–34

☐ 35–44

☐ 45–54

☐ 55+

*Gender*

☐ Male

☐ Female

☐ Non-binary

☐ Prefer not to say

☐ Other

*Highest level of education completed*

☐ No formal education

☐ High school

☐ Undergraduate degree

☐ Postgraduate degree

☐ Other

*Current setting of practice*

☐ Secular organisation

☐ Religious organisation

☐ Mixed secular and religious setting

☐ Work from home

☐ Other

*Religious affiliation*

☐ Protestantism

☐ Catholicism

☐ Christianity

☐ Judaism

☐ Islam

☐ Buddhism

☐ Hinduism

☐ Inter/Non-denominational

☐ No religion

☐ Other

*Sector of work*

☐ Healthcare

☐ Education

☐ Industry/Manufacturing

☐ Government/Public Service

☐ Non-profit/Charity

☐ Research/Academia

☐ Technology/IT

☐ Finance/Business

☐ Other

# 4 Section B. Experiences of Receiving Compassion

How often do you experience acts of compassion from others?

☐ Never

☐ Rarely

☐ Sometimes

☐ Often

☐ Always

What factors influence this? ________________________

# 5 Section C. Interpretation of Compassion

From which sources do you primarily derive your understanding of compassion received from others?

☐ Religious teachings

☐ Personal life experiences and common-sense reasoning

☐ Secular ethical frameworks

☐ Family influences

☐ Situational or professional settings

☐ Other

Share a personal example when you felt you received an act of compassion from others:

# 6 Section D. Attitudes Towards Receiving Compassion

Do you feel comfortable receiving compassion from others?

☐ Yes

☐ No

☐ Sometimes

Please explain: _________________________________

# 7 Section E. Compassion and Well-Being

To what extent does receiving compassion contribute to your quality of life?

☐ Not at all

☐ Slightly

☐ Moderately

☐ Significantly

☐ Extremely

Describe how compassion influences your mental well-being, coping ability, or overall quality of life:

# 8 Section F. Settings of Compassion

☐ Family

☐ Friends

☐ Workplace

☐ Religious or spiritual community

☐ Society at large

☐ Online or digital environments

☐ Other

# 9 Section G. Organizational Well-Being

To what extent does receiving compassion contribute to organizational well-being?

☐ Not at all

☐ Slightly

☐ Moderately

☐ Significantly

☐ Extremely
